# FEM1B enhances TRAIL‐induced apoptosis in T lymphocytes and monocytes

**DOI:** 10.1002/2211-5463.70056

**Published:** 2025-05-20

**Authors:** Chenbo Yang, Wenhui Yu, Cui Dang, Jingjing Zhang, Jiahan Lu, Jing Xue

**Affiliations:** ^1^ NHC Key Laboratory of Human Disease Comparative Medicine, State Key Laboratory of Respiratory Health and Multimorbidity, Beijing Key Laboratory for Animal Models of Emerging and Remerging Infectious Diseases, Institute of Laboratory Animal Science Chinese Academy of Medical Sciences and Peking Union Medical College Beijing China; ^2^ Center for AIDS Research Chinese Academy of Medical Sciences and Peking Union Medical College Beijing China

**Keywords:** apoptosis, caspase pathway, FEM1B, monocytes, T lymphocytes, TRAIL

## Abstract

FEM1B is recognized for its significant pro‐apoptotic function in colorectal cancer; however, its influence and mechanisms regarding apoptosis in immune cells remain inadequately elucidated. In this study, we demonstrated that FEM1B enhances TRAIL‐induced apoptosis in Molt‐4, Jurkat, THP‐1, and U937 cell lines. Notably, the knockdown of FEM1B in transfected cells resulted in a reversal of the observed increase in cell apoptosis. Our findings indicate that FEM1B activates caspase‐3 and caspase‐8, but not caspase‐9, in response to TRAIL stimulation, suggesting its involvement in the extrinsic caspase‐dependent apoptotic pathway. Furthermore, we found that FEM1B interacted with TRAF2 and downregulates its expression in Molt‐4 and Jurkat cells, thereby diminishing TRAF2's inhibitory effect on caspase‐8. In THP‐1 and U937 cells, FEM1B was found to upregulate TRAIL‐R2, thereby promoting TRAIL‐induced apoptosis. Knockout studies in murine models further corroborated that FEM1B facilitates TRAIL‐induced apoptosis. These results demonstrate that FEM1B enhances TRAIL‐induced apoptosis in T lymphocytes and monocytes through a caspase‐dependent mechanism involving TRAF2 or TRAIL receptors.

## Graphical abstract

FEM1B facilitates TRAIL‐induced apoptosis through distinct mechanisms in T lymphocytes and monocytes. In T lymphocytes, FEM1B engages with TRAF2, leading to a reduction in TRAF2 expression, which subsequently lessens TRAF2's inhibitory influence on caspase‐8. Conversely, in monocytes, FEM1B is observed to enhance the expression of TRAIL‐R2, which in turn promotes the activation of caspase‐3, thereby augmenting TRAIL‐induced apoptosis.

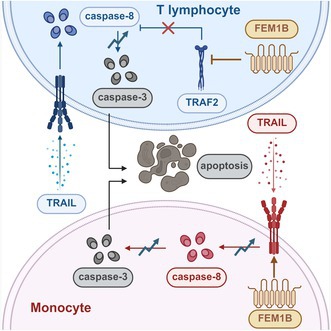

**Abbreviations**
ANOVA: analysis of variance
APAF1: apoptotic protease activating factor 1
ATCC: American Type Culture Collection
BAK: Bcl‐2 homologous antagonist killer
BAX: Bcl‐2 associated X
BID: BH3‐interacting domain death agonist
CRL2: cullin2‐ring ubiquitin ligase
FADD: Fas‐associated death domain
FEM1B: Fem‐1 homolog B
FNIP1: folliculin‐interacting protein 1
GAPDH: glyceraldehyde‐3‐phosphate dehydrogenase
PBS: phosphate‐buffered saline
qPCR: quantitative PCR
ROS: reactive oxygen species
SEM: standard error of mean
TNF: tumor necrosis factor
TRAF2: TNF receptor‐associated factor 2
TRAIL: TNF‐related apoptosis‐inducing ligand

Apoptosis is a fundamental physiological process characterized by programmed cell death, which is crucial for maintaining homeostasis and facilitating tissue development in adults [1, 2, 3]. Dysregulation in apoptosis, whether through inhibition or excessive activation, contributes to the pathogenesis of tumors, neurodegenerative disorders, and immune dysregulation [4, 5, 6, 7, 8]. Apoptosis can be triggered by pro‐apoptotic receptors on the cell membrane or through intracellular mechanisms involving mitochondria. The activation of the ICE/Ced‐3‐like protease cascade, commonly referred to as caspases, represents a common pathway that integrates various apoptotic signals [9, 10].

Tumor necrosis factor apoptosis‐inducing ligand (TRAIL) is classified within the type II membrane tumor necrosis factor (TNF) ligand family [11, 12]. Once TRAIL binds to its receptors (TRAIL‐R1 or TRAIL‐R2), Fas‐associated death domain (FADD) will be recruited to them, leading to the activation of caspase‐8 via proximity‐induced dimerization and proteolytic cleavage [13, 14]. Activated caspase‐8 can directly activate caspase‐3 to drive apoptosis or induce the cleavage of BH3‐interacting domain death agonist (BID) and generate truncated form (tBID), which interacts with B‐cell lymphoma 2 (Bcl‐2)‐associated X (BAX) and Bcl‐2 homologous antagonist killer (BAK) to execute mitochondrial outer membrane permeabilization and the release of cytochrome C, leading to the assembling of intracellular initiator caspase‐9 [15, 16], thereby amplifying the apoptotic signaling cascade [17, 18].

Tumor necrosis factor receptor‐associated factor 2 (TRAF2) functions as an intracellular adaptor that plays a critical role in the negative regulation of apoptosis. TRAF2 is capable of directly participating in the ubiquitination of caspase‐8, thereby contributing to its antiapoptotic properties [19]. Additionally, TRAF2 disrupts the interaction between FADD and caspase‐8, leading to the inactivation of caspase‐8 [20, 21]. The essential function of TRAF2 as a negative regulator of apoptosis is evidenced *in vivo* by the observation that TRAF2 knockout mice exhibit early postnatal mortality due to elevated serum levels of TNF [22, 23]. Furthermore, various studies utilizing TRAF2‐deficient cell lines demonstrate that the protective effects of TRAF2 against TNF‐induced apoptosis are applicable across multiple cell types [24]. FEM1B, a protein exhibiting significant homology to FEM‐1, which is essential for sex determination in *Caenorhabditis elegans* [25]. FEM1B is classified as a member of the FEM1 family, also including FEM1A and FEM1C, which is characterized as an ankyrin repeat protein family. This family is associated with death receptor‐related proteins and is involved in the regulation of apoptosis [26]. FEM1B has been shown to promote apoptosis when its expression levels are elevated in various cancer cell types, including those associated with breast cancer, cervical cancer, neuroblastoma, and fibrosarcoma [27].

In previous studies, it has been reported that FEM1B interacts with the intracellular death domains of apoptosis‐inducing cell surface receptors, such as Fas and tumor necrosis factor receptor 1 (TNFR1), as well as with apoptotic protease activating factor‐1 (APAF1) [28, 29]. Nevertheless, whether FEM1B evolves in the apoptotic pathway mediated by other death receptor family members and their ligands, and its role in TRAIL‐mediated apoptosis and the mechanisms by which FEM1B affects apoptosis in immune cells remain unclear, despite the close relationship among Fas, TNF, and TRAIL within the TNF superfamily. Additionally, the pro‐apoptotic function of FEM1B has yet to be validated in immune cells. In this study, we utilized T lymphocyte and monocyte cell lines, along with a FEM1B knockout murine model, to demonstrate that FEM1B can enhance TRAIL‐induced apoptosis in immune cells. We also explore the potential mechanism by which FEM1B enhances TRAIL‐induced apoptosis in T lymphocytes and monocytes.

## Materials and methods

### Cells and plasmids

Human 293T (ATCC; #CRL‐3216) cell line was cultured in Dulbecco's modified Eagle's medium (Gibco, New York, NY, USA). Human Molt‐4 (ATCC; #CRL‐1582), Human Jurkat (clone E6‐1; ATCC; #TIB‐152), Human THP‐1 (ATCC; #TIB‐202), and Human U937 (ATCC; #CRL‐1593.2) cell lines were cultured in Roswell Park Memorial Institute‐1640 medium (Gibco). All media were supplemented with 10% fetal bovine serum (Gibco), 100 U·mL^−1^ penicillin, and 100 mg·mL^−1^ streptomycin.

The open reading frames of the expression vectors encoding FEM1B and shFEM1B were cloned in pLV‐3flag‐P2A, the plasmid pSPAX2, pMD2.G based on lentiviral system were obtained from Addgene. Plasmids were transfected in 293T cells using lipoD293 (SignaGen, Frederick, MD, USA).

### qPCR

Total RNA was extracted utilizing the RNeasy® Mini kit in accordance with the manufacturer's guidelines (QIAGEN, Hilden, Germany). The reverse transcription process was conducted using the PrimeScript™ RT Reagent Kit with gRNA eraser (TaKaRa, Beijing, China). The gDNA eraser was applied to RNA samples and incubated at 42 °C for 2 min. Reverse transcription reaction was maintained at 37 °C for 15 min, followed by a brief incubation at 85 °C for 5 s. For real‐time PCR, primers were used, including: 5′‐GACACGCAAAGGTGGTACGC‐3′ and 5′‐GCTCCAGCTGCACACCAAAG‐3′ for FEM1B; 5′‐AATGACCCCTTCATTGAC‐3′ and 5′‐TCCACGACGTACTCAGCGC‐3′ for GAPDH. The qPCR protocol involved an initial denaturation at 95 °C for 30 s, followed by 40 cycles of denaturation at 95 °C for 5 s and annealing/extension at 62 °C for 34 s. qPCR result analysis was performed using the ΔΔCT method normalized to GAPDH.

### Immunoblotting

Immunoblotting was performed to detected cellular protein level using various antibodies, including rabbit anti‐FEM1B polyclonal (Thermo Fisher, Waltham, MA, USA, 1 : 500), rabbit anti‐caspase‐3 monoclonal (CST, Boston, MA, USA, 1 : 1000), rabbit anti‐cleaved caspase‐3 (CST, 1 : 1000), rabbit anti‐caspase‐8 monoclonal (CST, 1 : 1000), rabbit anti‐cleaved caspase‐8 (CST, 1 : 1000), mouse anti‐caspase‐9 monoclonal (CST, 1 : 1000), mouse anti‐cleaved caspase‐9 (CST, 1 : 1000), rabbit anti‐BAK monoclonal (CST, 1 : 500), rabbit anti‐BAX monoclonal (CST, 1 : 500), rabbit anti‐cytochrome C monoclonal (CST, 1 : 500), rabbit anti‐TRAF2 monoclonal (CST, 1 : 500), rabbit anti‐TRAIL‐R1 (CST, 1 : 500), rabbit anti‐TRAIL‐R2 (CST, 1 : 500), rabbit anti‐GAPDH (CST, 1 : 3000), HRP anti‐rabbit IgG (CST, 1 : 5000), and HRP anti‐mouse IgG (CST, 1 : 5000).

### Measurement of cell apoptosis

Cell apoptosis was measured by flow cytometry. Cells were subjected to a centrifuging wash procedure using precooled PBS, then cells were resuspended in 1× Binding Buffer (Sino Biological, Beijing, China). Five microliters of annexin V‐PE and 7‐AAD antibody (Sino Biological) were added to the cell suspension, which was then gently mixed and incubated for 15 min at room temperature. The fluorescence was monitored by BD Accuri™ C6. The resulting data were analyzed and visualized using flowjo software (version 10.0, provided by BD Biosciences, Franklin Lakes, NJ, USA).

### TRAIL inducement and caspase inhibitor assay

TRAIL human protein (MCE, New Jersey, USA) was applied in the concentration of 100 ng·mL^−1^ for 16 h. Caspase‐3 inhibitor Z‐DEVD‐FMK, caspase‐8 inhibitor Z‐IETD‐FMK, and pan‐caspase inhibitor Z‐VAD‐FMK (MCE) were applied in the concentration of 50 nm and used 1 h prior to TRAIL inducement.

### Co‐immunoprecipitation

Human 293T cells were collected 48 h post‐transfection. Subsequently, 5 μg of mouse anti‐Flag antibody was co‐incubated with protein A agarose beads and protein G agarose beads overnight at 4 °C. Immunoblotting was subsequently employed to identify the binding proteins through co‐immunoprecipitation.

### Animal care and study approval

The experimental protocol was approved by the Institutional Animal Care and Use Committee (IACUC, approval number: XJ23007) at the Institute of laboratory animal science (ILAS), CAMS&PUMC. All animal care procedures adhered to the guidelines for the ethical treatment and use of animals. FEM1B‐KO mice (C57BL/6) were procured from ILAS. Heterozygous male and female mice were mated to produce offspring. Six‐ to eight‐week‐old mice were euthanized using spinal cord dislocation method. A tail biopsy was performed on the newborn mice at postnatal Day 7 for DNA extraction and PCR analysis using primer 5′‐TCAACCTTCCTAATGCTGCAACT‐3′ and 5′‐CATTCCTTTTCATCTTACACCTAATTAG‐3′ for FEM1B KO verification. Experimental procedures were conducted using homozygous KO mice alongside their WT littermates.

### Assessment cell apoptosis in murine spleen cells

Mice spleens were excised and gently grinded using 70 μm cell strainer to obtain single‐cell suspension. Mouse TRAIL (MCE) was then applied for 16 h in the concentration of 100 ng·mL^−1^. Anti‐CD3‐BV510 and CD11b‐FITC antibodies (BD Biosciences) were incorporated into the cell suspension, and incubated for 30 min at 4 °C, Annexin V and 7‐AAD antibodies were then added and incubated for 15 min at room temperature to test *in vitro* cell apoptosis in T lymphocytes and monocytes with or without TRAIL inducement. The fluorescence was monitored by BD FACS Arial™ Fusion II.

## Results

### FEM1B does not affect noninduced cell apoptosis

**Fig. 1:**
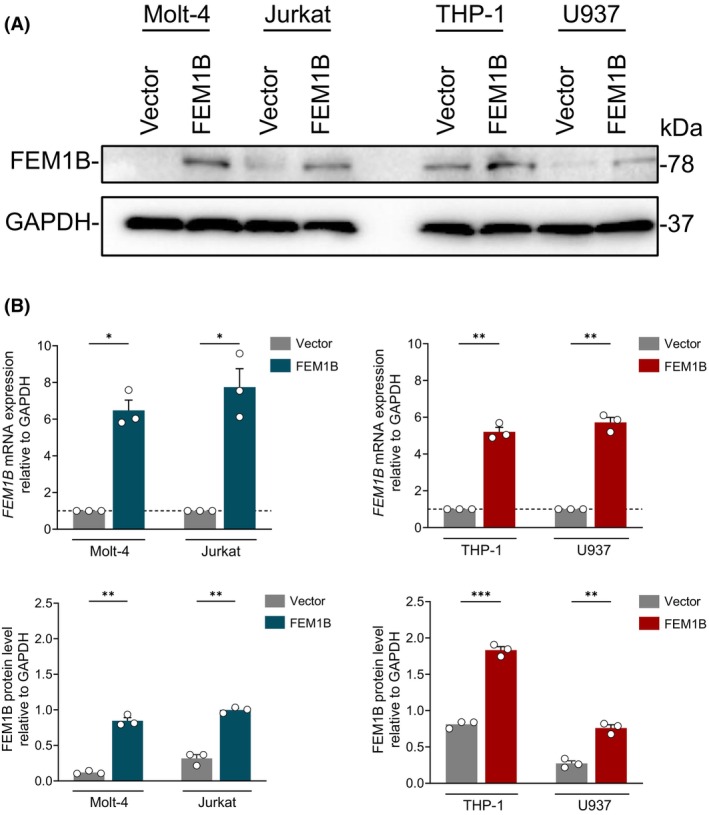
Overexpression of FEM1B in cell lines derived from T lymphocytes and monocytes. (A) The protein level of FEM1B in both transfected and mock Molt‐4, Jurkat, THP‐1, and U937 cell lines, as assessed through immunoblotting. (B) FEM1B mRNA expression of transfected and nontransfected cell lines using quantitative polymerase chain reaction (qPCR) (upper panel) and FEM1B protein level in statistical representation (lower panel), with statistical significance indicated at *P < 0.05, **P < 0.01, ***P < 0.001, analyzed by Welch's *t*‐test. Error Bars represent mean ± SEM, *n* = 3 technical replicates.

**Fig. 2:**
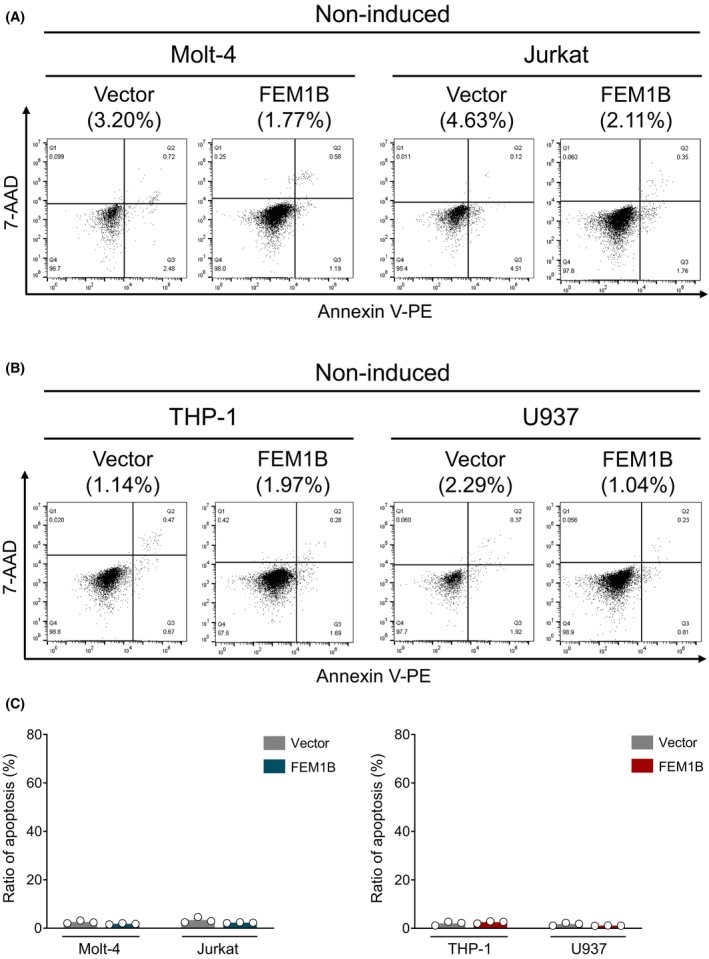
Occurrence of noninduced cell apoptosis in both transfected and mock (A) Molt‐4 and Jurkat cell lines, (B) THP‐1 and U937 cell lines, as well as the (C) statistical representation of the data; statistical significance is analyzed by Welch's *t*‐test, *n* = 3 technical replicates.

We utilized Molt‐4 and Jurkat cell lines as models for T lymphocytes, and THP‐1 and U937 cell lines for monocytes. Human *FEM1B* was transfected into these cell lines using a lentivirus system, with subsequent protein and mRNA levels assessed by immunoblotting and quantitative PCR (qPCR) (Fig. 1A,B). Transfection resulted in a significant increase in both FEM1B protein level and mRNA expression compared with mock cells, confirming successful overexpression across all cell lines. To figure out whether FEM1B transfection alone affects cell apoptosis, we compared the noninduced cell apoptosis in FEM1B transfected cells to those in mock cells. In T lymphocytes, FEM1B did not alter noninduced cell apoptosis rates in either Molt‐4 cells (1.80 ± 0.246% vs. 2.56 ± 0.58% in mock cells) or Jurkat cells (2.28 ± 0.17% vs. 3.35 ± 1.13% in mock cells) (Fig. 2A,C). Similarly, FEM1B transfected THP‐1 and U937 monocyte cells showed cell apoptosis of 2.54 ± 0.51% and 1.09 ± 0.08%, respectively (Fig. 2B,C), indicating no significant impact of FEM1B on noninduced cell apoptosis. These results suggest that FEM1B may not influence cell apoptosis in T lymphocyte or monocyte cell lines without any inducement.

### FEM1B enhances TRAIL‐induced cell apoptosis

**Fig. 3:**
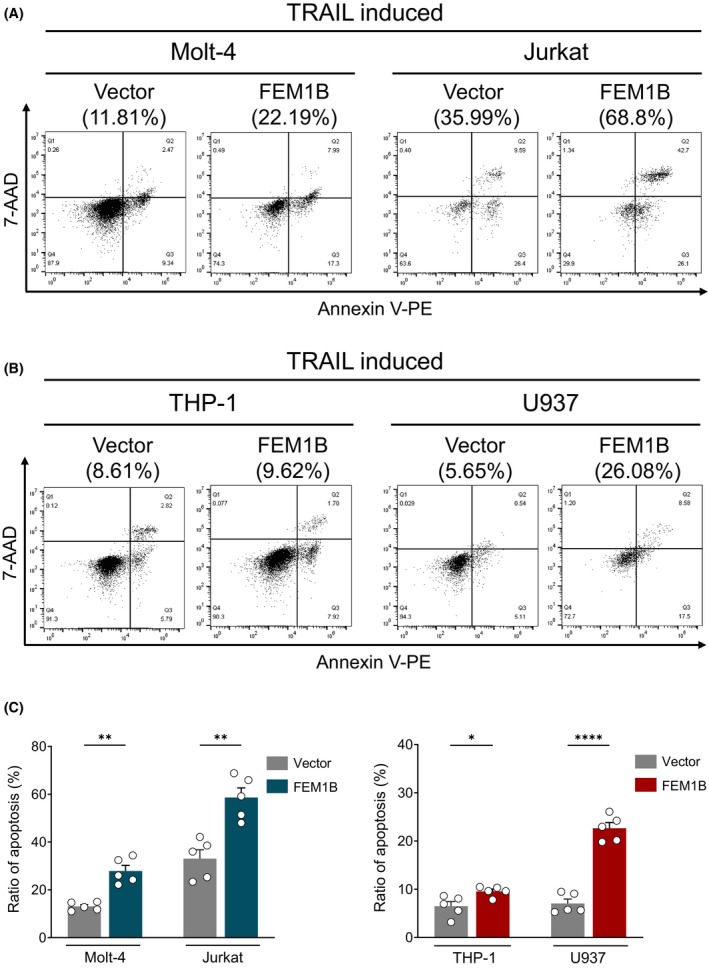
Cell apoptosis induced by TRAIL in both transfected and mock (A) Molt‐4 and Jurkat cell lines, (B) THP‐1 and U937 cell lines, as well as the (C) statistical representation of the data. Statistical significance indicated at *P < 0.05, **P < 0.01, ****P < 0.0001, analyzed by Welch's *t*‐test. Error Bars represent mean ± SEM, *n* = 5 technical replicates.

To further confirm the role of FEM1B in cell apoptosis, we measured cell apoptosis induced by TRAIL, which acting as a blasting fuse to trigger apoptosis. In the Jurkat cells, FEM1B significantly increased cell apoptosis induced by TRAIL, with ratio of 58.63 ± 8.98% compared with 32.87 ± 8.21% in mock cells. Similarly, in Molt‐4 cell lines, cell apoptosis raised up to 27.85 ± 5.30% with FEM1B transfection, compared with 13.07 ± 1.75% in mock cells (Fig. 3A,C). In cell lines derived from monocytes, FEM1B also enhanced cell apoptosis, with ratio of 9.57 ± 1.05% in THP‐1 cells versus 6.47 ± 2.13% in mock cells, and 22.65 ± 2.68% in U937 cells compared with 7.04 ± 2.05% in mock cells (Fig. 3B,C). Although the extent of apoptosis varied among different cell lines, FEM1B significantly increased their sensitivity to TRAIL, resulting in promoted cell apoptosis across various cell lines, particularly in T lymphocytes. Notably, Jurkat cells exhibited a more pronounced increase in apoptosis in response to TRAIL inducement compared with other cell lines, which may be attributed to their heightened sensitivity toward TRAIL inducement.

**Fig. 4:**
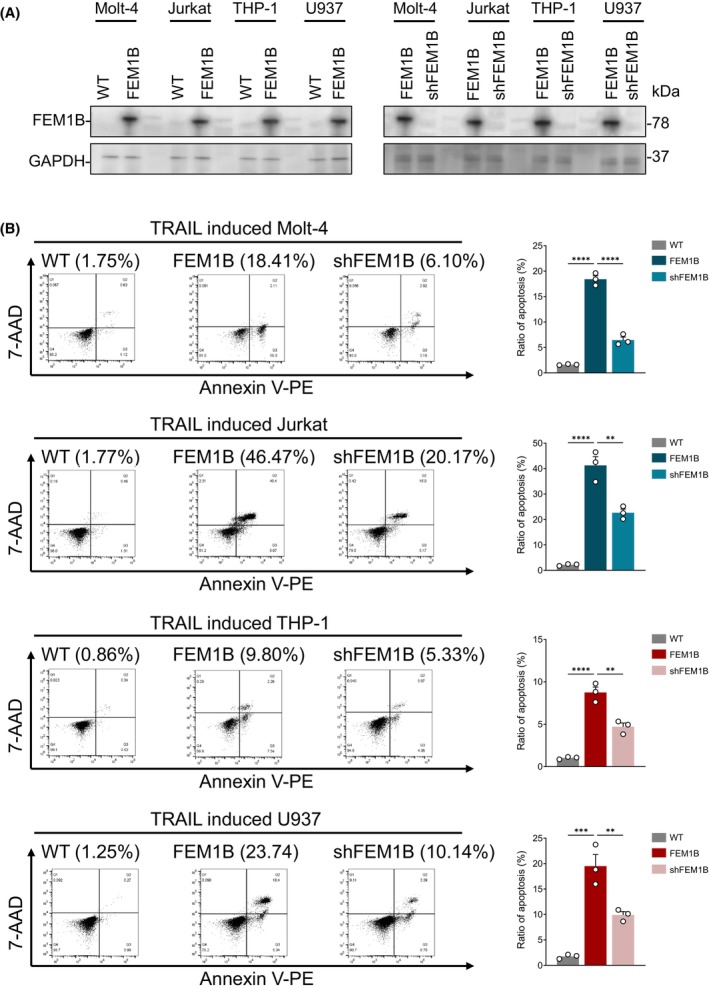
Rescue experiment of FEM1B overexpression. (A) Examination of FEM1B protein level in transfected and nontransfected Molt‐4, Jurkat, THP‐1, and U937 cell lines (left panel) and FEM1B depletion in transfected cell lines (right panel). (B) TRAIL‐induced cell apoptosis in nontransfected, FEM1B transfected, and FEM1B depletion in transfected Molt‐4, Jurkat, THP‐1, and U937 cell lines as well as the statistical representation of the data. Statistical significance indicated at **P < 0.01, ****P < 0.0001, analyzed by ordinary one‐way ANOVA and Dunnett's multiple comparisons test. Error Bars represent mean ± SEM, *n* = 3 technical replicates.

To ascertain the role of FEM1B in TRAIL‐induced cell apoptosis and to further substantiate their relationship, we conducted a rescue experiment. We selectively depleted FEM1B in cell lines that had been transfected with FEM1B and subsequently compared the levels of TRAIL‐induced apoptosis in these modified cell lines to those in wild‐type (WT) and nondepleted counterparts (Fig. 4A). Our findings indicated that the downregulation of FEM1B can partially reverse the increased apoptosis associated with its overexpression. Specifically, in Molt‐4 and Jurkat cells, the ratio of apoptosis was reduced to 6.46 ± 1.03% and 22.64 ± 2.51%, respectively. In contrast, THP‐1 and U937 cells exhibited apoptosis rates of 6.46 ± 1.03% and 9.90 ± 1.15% (Fig. 4B). These results support the hypothesis that FEM1B plays a significant role in mediating TRAIL‐induced apoptosis.

### FEM1B enhanced cell apoptosis associates with the activation of caspase‐3 and caspase‐8

**Fig. 5:**
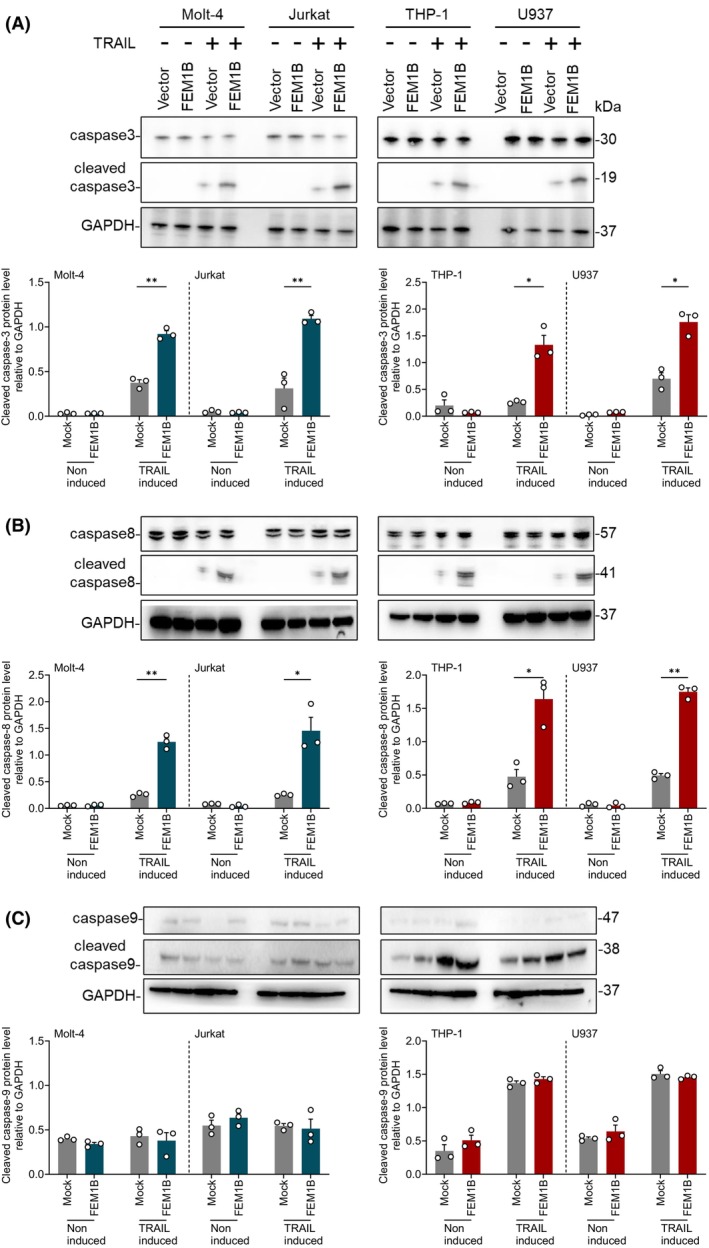
Protein level of caspase molecules and their cleaved forms in the caspase‐dependent pathway in FEM1B transfected and mock Molt‐4, Jurkat, THP‐1, and U937 cell lines. (A) The protein levels of caspase‐3 along with their cleaved forms and the statistical representation of cleaved caspase‐3. (B) The protein levels of caspase‐8 along with their cleaved forms and the statistical representation of cleaved caspase‐8. (C) The protein levels of caspase‐9 along with their cleaved forms and the statistical representation of cleaved caspase‐9. Statistical significance indicated at *P < 0.05, **P < 0.01, analyzed by Welch's *t*‐test. Error Bars represent mean ± SEM, *n* = 3 technical replicates.

**Fig. 6:**
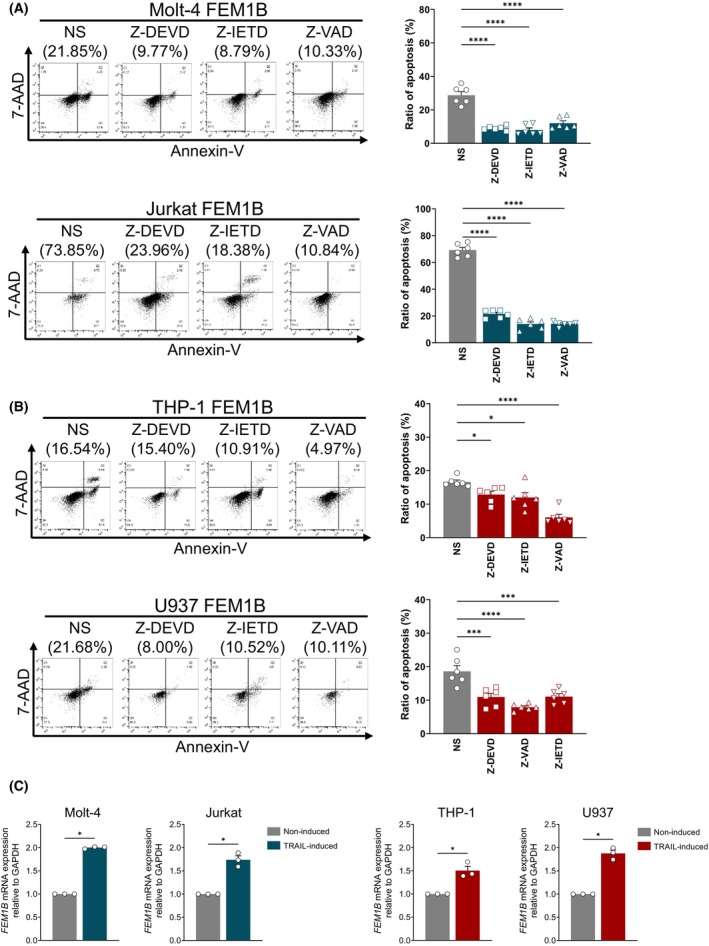
Effect of a caspase inhibitor on the reduction of apoptosis induced by FEM1B transfection and the FEM1B expression related to apoptosis in Molt‐4, Jurkat, THP‐1, and U937 cell lines. (A) Data from Molt‐4 and Jurkat cell lines transfected with FEM1B, which were subjected to treatment with saline, Z‐DEVD, Z‐IETD, and Z‐VAD, yielding a statistically significant result of ****P < 0.0001. (B) Findings from transfected THP‐1 and U937 cell lines treated with saline, Z‐DEVD, Z‐IETD, and Z‐VAD, revealing significant differences with *P < 0.05, ***P < 0.001, and ****P < 0.0001. Ordinary one‐way ANOVA and Dunnett's multiple comparisons test were used for statistical analysis. Error Bars represent mean ± SEM, *n* = 6 technical replicates. (C) The FEM1B mRNA expression in noninduced or TRAIL‐induced Molt‐4, Jurkat, THP‐1 and U937 cell lines. Statistical significance indicated at *P < 0.05, analyzed by Welch's *t*‐test. Error Bars represent mean ± SEM, *n* = 3 technical replicates.

To investigate how FEM1B affects cell apoptosis induced by TRAIL, we examined the caspase‐dependent pathway, representing the classic downstream signaling triggered by TRAIL and its receptor. In the absence of TRAIL, caspase‐3, caspase‐8, and caspase‐9 remained in their un‐cleaved forms in FEM1B transfected and mock Molt‐4, Jurkat, THP‐1, and U937 cells. However, following TRAIL inducement, the level of caspase cleavage was significantly increased. We observed that upon TRAIL inducement, the cleavage forms of caspase‐3 and caspase‐8 were significantly elevated in FEM1B transfected Molt‐4, Jurkat, THP‐1, and U937 cells, while caspase‐9 was also cleaved upon inducement but showed no significance between transfected and mock cells (Fig. 5A–C). We further confirmed that Z‐DEVD‐FMK (a caspase‐3 inhibitor), Z‐IETD‐FMK (a caspase‐8 inhibitor), and Z‐VAD‐FMK (a pan‐caspase inhibitor) were able to block TRAIL‐induced apoptosis (Fig. 6A,B). In addition, we detected FEM1B expression in Molt‐4, Jurkat, THP‐1, and U937 cells with or without TRAIL inducement (Fig. 6C); our findings indicated that FEM1B demonstrated increased expression level in response to TRAIL induction. Consequently, we hypothesize that FEM1B may function within a caspase‐dependent pathway and possess the capacity to respond to TRAIL, leading to its upregulation.

**Fig. 7:**
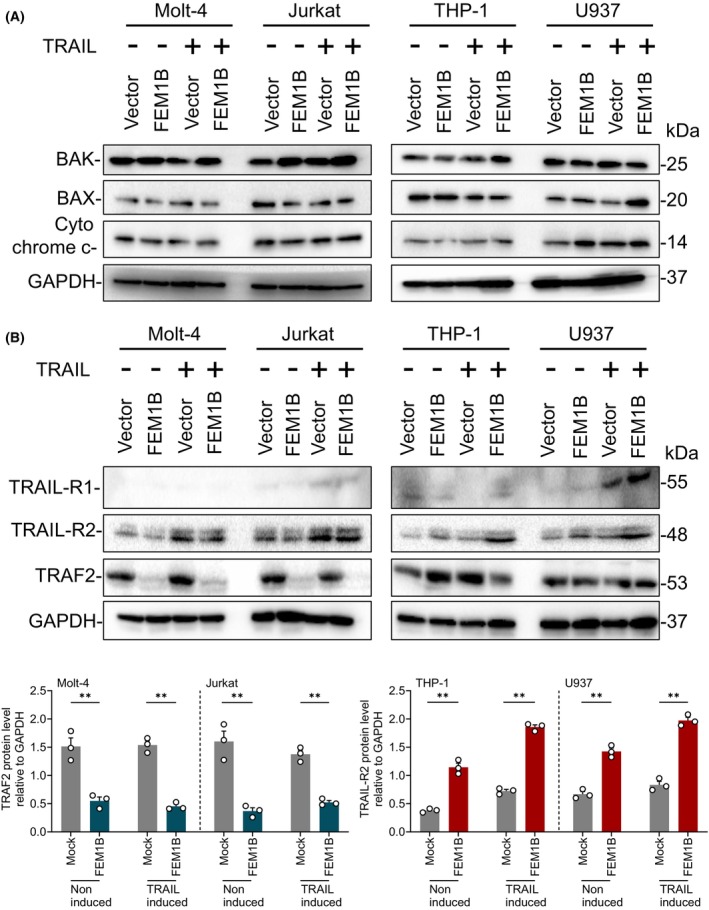
Protein level of apoptosis‐related molecules in Molt‐4, Jurkat, THP‐1, and U937 cell lines. (A) The protein level of BAK, BAX, and cytochrome C. (B) The protein level of TRAIL‐R1, TRAIL‐R2, and TRAF2, and the statistical presentation of TRAF2 protein level in Molt‐4 and Jurkat cell lines, as well as TRAIL‐R1 protein level in THP‐1 and U937 cell lines. Statistical significance indicated at **P < 0.05, analyzed by Welch's *t*‐test. Error Bars represent mean ± SEM, *n* = 3 technical replicates.

Furthermore, we assessed the protein levels of intrinsic apoptotic molecules, including BAK, BAX, and cytochrome C, but we did not observe any significant change in these molecules (Fig. 7A).

### FEM1B deploys TRAF2 and TRAIL‐R2 to mediate apoptosis

**Fig. 8:**
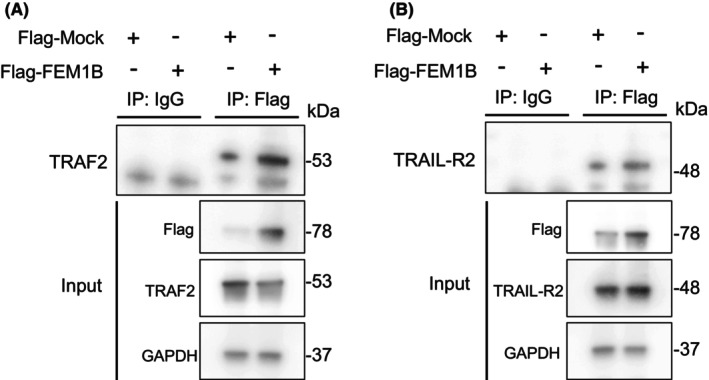
Interaction of FEM1B with TRAF2 and TRAIL‐R2. (A) 293T cells transfected with the FEM1B‐flag plasmid were subjected to anti‐Flag immunoprecipitation to test the interaction between FEM1B and TRAF2. (B) 293T cells transfected with the FEM1B‐flag plasmid were subjected to anti‐Flag immunoprecipitation to test the interaction between FEM1B and TRAIL‐R2, analyzed by immunoblotting.

To explore the specific function of FEM1B in caspase‐dependent cellular apoptosis, we conducted an analysis of the protein levels of TRAIL receptors, specifically TRAIL‐R1, TRAIL‐R2, and the receptor‐associated factor TRAF2. Our findings indicated that the expression profiles of TRAIL‐R1, TRAIL‐R2, and TRAF2 varied in response to FEM1B transfection. In Molt‐4 and Jurkat cell lines, TRAIL‐R1 was undetectable, and the expression level of TRAIL‐R2 did not exhibit a significant difference between transfected and mock cells. Notably, we observed a significant decrease in TRAF2 levels in FEM1B‐transfected cells, regardless of induction status (Fig. 7B). In THP‐1 and U937 cell lines, TRAIL‐R1 levels were marginally elevated compared with those in Molt‐4 and Jurkat cells. However, both TRAIL‐R1 and TRAF2 did not show significant differences between transfected and mock cells. Notably, FEM1B‐transfected THP‐1 and U937 cells demonstrated an increased protein level of TRAIL‐R2 compared with their mock counterparts (Fig. 7B). Furthermore, we established that FEM1B interacts with TRAF2 (Fig. 8A) but not with TRAIL‐R2 (Fig. 8B), leading us to hypothesize that TRAF2 may serve as a target for FEM1B, particularly in the context of ubiquitination. Nonetheless, the precise mechanism by which FEM1B influences TRAIL‐R2 remains to be clarified.

### FEM1B gene knockout reduces TRAIL‐induced apoptosis in splenic cells

**Fig. 9:**
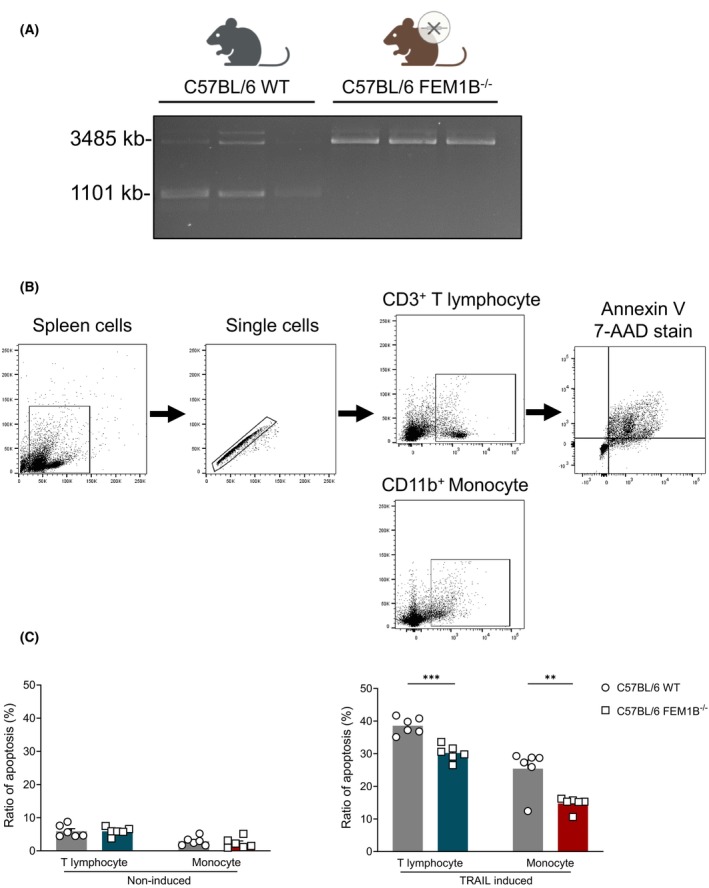
Knockout of the FEM1B gene mitigates TRAIL‐induced apoptosis in T lymphocytes and monocytes separated from murine spleens. (A) Tail biopsies from FEM1B knockout (KO) mice and their wild‐type (WT) littermates were analyzed using polymerase chain reaction (PCR). (B) The gating strategy employed for the identification of CD3^+^ T lymphocytes and CD11b^+^ monocytes from splenic cells, along with the subsequent analysis of cell apoptosis, is presented. (C) The proportion of noninduced (left panel) and TRAIL‐induced cell apoptosis within the CD3^+^ T lymphocytes and CD11b^+^ monocytes population from FEM1B‐KO mice compared with WT littermates is depicted. Statistical significance is indicated by **P < 0.01, ***P < 0.001, analyzed by Welch's *t*‐test. Error Bars represent mean ± SEM, *n* = 6 biological replicates.

To investigate the impact of FEM1B knockout on TRAIL‐induced cell apoptosis *in vivo*, we conducted a knockout of FEM1B in C57BL/6 mice (Fig. 9A). The spleens of these mice were processed into a single‐cell suspension (Fig. 9B), with some samples subjected to Mouse TRAIL treatment and others left untreated. T lymphocytes and monocytes from FEM1B knockout and WT mice did not undergo apoptosis in the absence of TRAIL stimulation. However, when exposed to TRAIL, these cells from FEM1B knockout mice exhibited a significant reduction in TRAIL‐induced apoptosis (Fig. 9C). These results contribute to a deeper understanding of the critical role of FEM1B in the regulation of cell apoptosis and suggest that FEM1B may serve as a promising therapeutic target for the modulation of apoptotic pathways.

## Discussion

FEM1B is a recently discovered gene associated with various diseases. It has been shown to facilitate the degradation of the glioma‐associated oncogene Gli1, which is linked to the development of tolerance to oxaliplatin [30]. Within the ubiquitin‐proteasome system, FEM1B plays a crucial role as a component of E3 ubiquitin ligases [31]. Recent studies demonstrate that FEM1B forms a cullin2‐ring ubiquitin ligase (CRL2) complex, participating in the degradation of folliculin‐interacting protein 1 (FNIP1) and playing a significant role in the cellular response to reductive stress [32, 33]. Moreover, the polyubiquitination and subsequent protein turnover mediated by CRL2^FEM1B^ are contingent upon interactions between FEM1B‐degron and the dimerization state of the E3 ligase complex [34]. The inhibition of FEM1B within the FEM1B‐FNIP1 axis has been shown to decrease mitochondrial reactive oxygen species (ROS), thereby promoting angiogenesis [35, 36]. The involvement of FEM1B in the process of ubiquitination has been elucidated; however, its role in cell apoptosis remains inadequately documented. Although FEM1B is recognized as a pro‐apoptotic protein, its direct role in promoting cell apoptosis has not yet been clarified.

In this study, we utilized FEM1B transfected Molt‐4, Jurkat, THP‐1, and U937 cells to explore the relationship between FEM1B and cell apoptosis. We found that FEM1B has the ability to enhance TRAIL‐induced apoptosis. Research on TRAIL has advanced significantly, particularly concerning its antitumor pathways and therapeutic potential [37, 38, 39]. Beyond cancer treatment, nevertheless, TRAIL's pro‐apoptotic properties and widespread expression suggest broader roles, particularly in cell apoptosis regulation and homeostasis [17, 40]. A recent study has identified a novel mechanism of cell communication [41], revealing that the endothelium serves as a significant source of TRAIL in healthy circulation, which is compromised in cases of peripheral artery disease. The deletion of TRAIL was found to inhibit neo‐angiogenesis, pericyte recruitment, and vessel stabilization, ultimately leading to diminished blood perfusion in the lower limbs affected by ischemia [42]. Additionally, the expression of TRAIL in interferon responsive CD4^+^ helper (Th) cells and regulatory cells has the potential to impede the activation of Th cells, thereby shedding light on a phenomenon associated with allergic responses [43]. Moreover, tumor‐associated macrophages enriched with TIM3 and VISTA were observed to induce the death of cancer cells through TRAIL signaling [44].

**Fig. 10:**
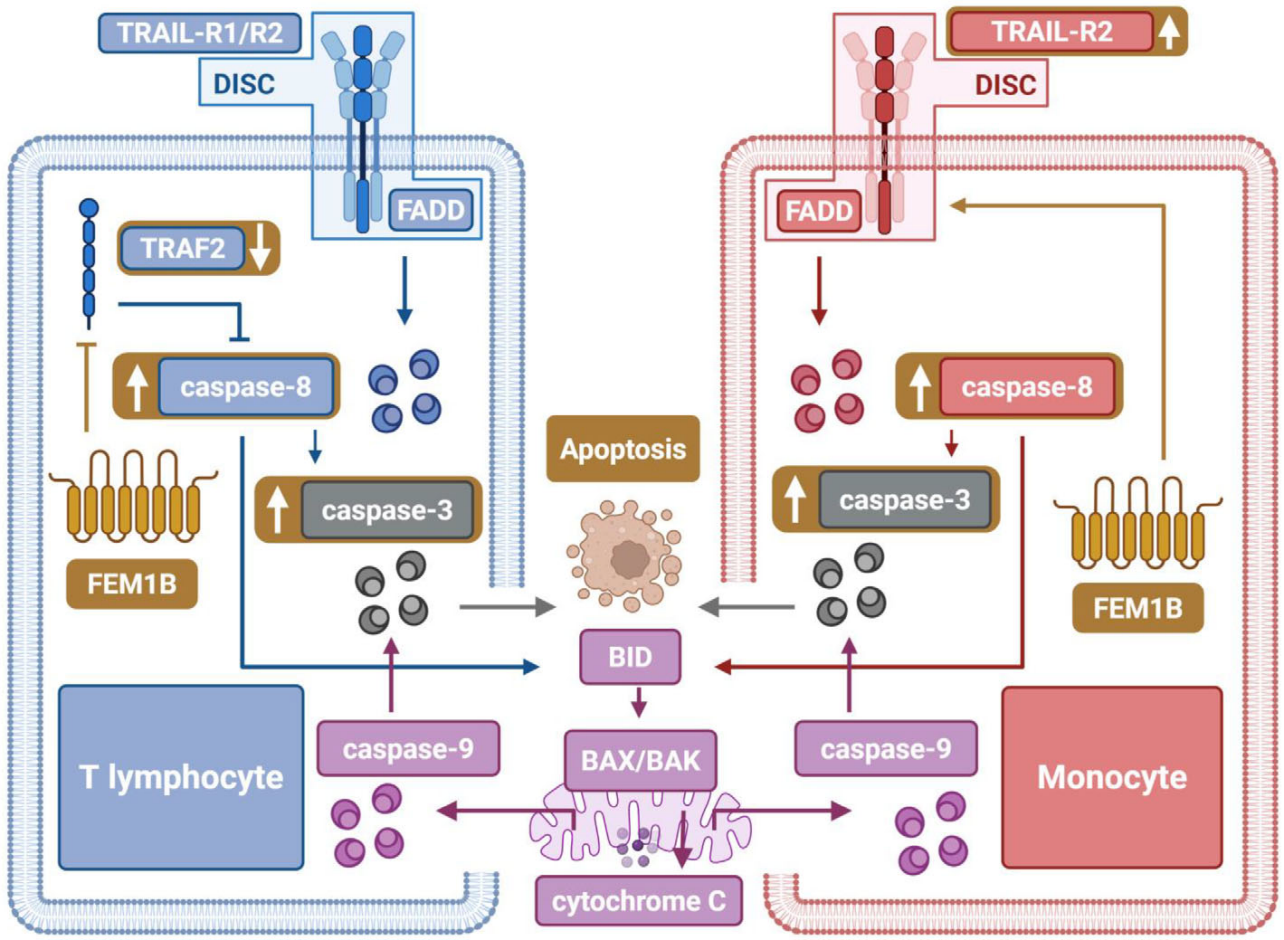
Illustration of how FEM1B facilitates TRAIL‐induced apoptosis in T lymphocytes and monocytes. In T lymphocytes, FEM1B downregulates TRAF2 expression, diminishing the inhibitory effects on caspase‐8. In monocytes, FEM1B enhances the expression of the death receptors TRAIL‐R2, promoting apoptotic processes.

Furthermore, we established a correlation between TRAIL‐induced apoptosis and FEM1B, demonstrating that FEM1B enhances TRAIL‐mediated apoptotic processes. Furthermore, we confirmed that the depletion of FEM1B can reverse the increased apoptosis associated with FEM1B overexpression, thereby affirming the essential role of FEM1B in promoting cellular apoptosis. Our analysis of downstream signaling pathways revealed the activation of caspases, which are integral to the classical TRAIL‐mediated pathway. Notably, FEM1B transfection resulted in a significant increase in the activation of caspase‐3 and caspase‐8, while having no effect on caspase‐9 activation. This suggests that FEM1B may specifically enhance the extrinsic apoptosis pathway, as it did not alter the levels of intrinsic apoptotic proteins, such as BAK, BAX, and cytochrome C. Importantly, the expression of FEM1B was found to be upregulated during apoptosis. In cell lines, such as Molt‐4, Jurkat, THP‐1, and U937, FEM1B is typically expressed at low levels; however, its upregulation in response to apoptotic stimuli provides a robust basis for its involvement in apoptotic processes. Our results further indicate that FEM1B modulates apoptosis in T lymphocyte and monocyte‐derived cell lines through various molecular pathways. In Molt‐4 and Jurkat cells, FEM1B was observed to downregulate TRAF2 expression, thereby reducing its inhibitory effects on caspase‐8. Conversely, in THP‐1 and U937 cells, FEM1B appeared to enhance the expression of the death receptor TRAIL‐R2, thereby enhancing the ligand‐receptor effect and facilitating TRAIL‐induced apoptotic processes (Fig. 10). We confirmed a relationship between TRAF2 and FEM1B, but not with TRAIL‐R2. Consequently, we propose that FEM1B may be involved in the degradation of TRAF2 due to its function as a ubiquitin ligase, although further evidence is required to confirm this hypothesis. Nonetheless, the precise mechanisms by which FEM1B influences apoptosis in THP‐1 and U937 cells remain to be elucidated. *In vivo*, we also showed that FEM1B knockout is able to dampen murine splenic cell apoptosis induced by TRAIL, further demonstrating its importance in TRAIL‐dependent cell apoptosis. The degradation of TRAF2, facilitated by FEM1B, plays a crucial role in moderating excessive immune responses, thereby helping to prevent autoimmunity. Additionally, through its interaction with TRAF2, FEM1B may also impact the NK‐κB signaling pathway, which is involved in various immune‐related disorders.

Over the past few decades, an increasing number of ligands and receptors within the TNF superfamily have been identified, expanding the understanding of programmed cell death beyond apoptosis to include mechanisms, such as necroptosis, pyroptosis, ferroptosis, mitotic catastrophe, and autophagic cell death [2, 45, 46, 47]. Collectively, we identified FEM1B as a novel enhancer of caspase‐3 and caspase‐8 cleavage, promoting increased cell death in response to TRAIL. Notably, the interaction between TRAIL and FEM1B appears to amplify the apoptotic effects of TRAIL alone. Future research is necessary to determine whether FEM1B is involved in other forms of programmed cell death, either in a similar or distinct manner, and to elucidate how FEM1B interacts with molecules within cell death signaling pathways.

## Conflict of interest

The authors declare no conflict of interest.

## Peer review

The peer review history for this article is available at https://www.webofscience.com/api/gateway/wos/peer‐review/10.1002/2211‐5463.70056.

## Author contributions

JX conceived and designed the experiments and revised the article. CY, WY, CD, JZ, ZC, and JL performed the experiments. CY analyzed the data and wrote the article. All authors have read and approved the article.

## Data Availability

The data that support the findings of this study are available from the corresponding author (xuejing@cnilas.org) upon reasonable request.

## References

[1] Carneiro BA and El‐Deiry WS (2020) Targeting apoptosis in cancer therapy. Nat Rev Clin Oncol 17, 395–417. 32203277 10.1038/s41571-020-0341-y PMC8211386

[2] Bertheloot D , Latz E and Franklin BS (2021) Necroptosis, pyroptosis and apoptosis: an intricate game of cell death. Cell Mol Immunol 18, 1106–1121. 33785842 10.1038/s41423-020-00630-3 PMC8008022

[3] Newton K , Strasser A , Kayagaki N and Dixit VM (2024) Cell death. Cell 187, 235–256. 38242081 10.1016/j.cell.2023.11.044

[4] Moyer A , Tanaka K and Cheng EH (2025) Apoptosis in cancer biology and therapy. Annu Rev Pathol 20, 303–328. 39854189 10.1146/annurev-pathmechdis-051222-115023

[5] Moujalled D , Strasser A and Liddell JR (2021) Molecular mechanisms of cell death in neurological diseases. Cell Death Differ 28, 2029–2044. 34099897 10.1038/s41418-021-00814-y PMC8257776

[6] Nowell J , Blunt E , Gupta D and Edison P (2023) Antidiabetic agents as a novel treatment for Alzheimer's and Parkinson's disease. Ageing Res Rev 89, 101979. 37328112 10.1016/j.arr.2023.101979

[7] Hanggi K and Ruffell B (2023) Cell death, therapeutics, and the immune response in cancer. Trends Cancer 9, 381–396. 36841748 10.1016/j.trecan.2023.02.001 PMC10121860

[8] Ren Y , Wang R , Weng S , Xu H , Zhang Y , Chen S , Liu S , Ba Y , Zhou Z , Luo P *et al*. (2023) Multifaceted role of redox pattern in the tumor immune microenvironment regarding autophagy and apoptosis. Mol Cancer 22, 130. 37563639 10.1186/s12943-023-01831-w PMC10413697

[9] Kesavardhana S , Malireddi RKS and Kanneganti TD (2020) Caspases in cell death, inflammation, and Pyroptosis. Annu Rev Immunol 38, 567–595. 32017655 10.1146/annurev-immunol-073119-095439 PMC7190443

[10] Yuan J and Ofengeim D (2024) A guide to cell death pathways. Nat Rev Mol Cell Biol 25, 379–395. 38110635 10.1038/s41580-023-00689-6

[11] Deng D and Shah K (2020) TRAIL of Hope meeting resistance in cancer. Trends Cancer 6, 989–1001. 32718904 10.1016/j.trecan.2020.06.006 PMC7688478

[12] Govindasamy B , Muthu M , Gopal J and Chun S (2023) A review on the impact of TRAIL on cancer signaling and targeting via phytochemicals for possible cancer therapy. Int J Biol Macromol 253, 127162. 37788732 10.1016/j.ijbiomac.2023.127162

[13] Montinaro A and Walczak H (2023) Harnessing TRAIL‐induced cell death for cancer therapy: a long walk with thrilling discoveries. Cell Death Differ 30, 237–249. 36195672 10.1038/s41418-022-01059-z PMC9950482

[14] Thapa B , Kc R and Uludag H (2020) TRAIL therapy and prospective developments for cancer treatment. J Control Release 326, 335–349. 32682900 10.1016/j.jconrel.2020.07.013

[15] Vandenabeele P , Bultynck G and Savvides SN (2023) Pore‐forming proteins as drivers of membrane permeabilization in cell death pathways. Nat Rev Mol Cell Biol 24, 312–333. 36543934 10.1038/s41580-022-00564-w

[16] Roberts AW , Wei AH and Huang DCS (2021) BCL2 and MCL1 inhibitors for hematologic malignancies. Blood 138, 1120–1136. 34320168 10.1182/blood.2020006785

[17] Oh YT and Sun SY (2021) Regulation of cancer metastasis by TRAIL/death receptor signaling. Biomolecules 11, 499. 33810241 10.3390/biom11040499 PMC8065657

[18] Maji A , Paul A , Sarkar A , Nahar S , Bhowmik R , Samanta A , Nahata P , Ghosh B , Karmakar S and Kumar Maity T (2024) Significance of TRAIL/Apo‐2 ligand and its death receptors in apoptosis and necroptosis signalling: implications for cancer‐targeted therapeutics. Biochem Pharmacol 221, 116041. 38316367 10.1016/j.bcp.2024.116041

[19] Gonzalvez F , Lawrence D , Yang B , Yee S , Pitti R , Marsters S , Pham VC , Stephan JP , Lill J and Ashkenazi A (2012) TRAF2 sets a threshold for extrinsic apoptosis by tagging caspase‐8 with a ubiquitin shutoff timer. Mol Cell 48, 888–899. 23142077 10.1016/j.molcel.2012.09.031

[20] van Loo G and Bertrand MJM (2023) Death by TNF: a road to inflammation. Nat Rev Immunol 23, 289–303. 36380021 10.1038/s41577-022-00792-3 PMC9665039

[21] Siegmund D and Wajant H (2023) TNF and TNF receptors as therapeutic targets for rheumatic diseases and beyond. Nat Rev Rheumatol 19, 576–591. 37542139 10.1038/s41584-023-01002-7

[22] Nguyen LT , Duncan GS , Mirtsos C , Ng M , Speiser DE , Shahinian A , Marino MW , Mak TW , Ohashi PS and Yeh WC (1999) TRAF2 deficiency results in hyperactivity of certain TNFR1 signals and impairment of CD40‐mediated responses. Immunity 11, 379–389. 10514016 10.1016/s1074-7613(00)80113-2

[23] Yeh WC , Shahinian A , Speiser D , Kraunus J , Billia F , Wakeham A , de la Pompa JL , Ferrick D , Hum B , Iscove N *et al*. (1997) Early lethality, functional NF‐kappaB activation, and increased sensitivity to TNF‐induced cell death in TRAF2‐deficient mice. Immunity 7, 715–725. 9390694 10.1016/s1074-7613(00)80391-x

[24] Borghi A , Verstrepen L and Beyaert R (2016) TRAF2 multitasking in TNF receptor‐induced signaling to NF‐kappaB, MAP kinases and cell death. Biochem Pharmacol 116, 1–10. 26993379 10.1016/j.bcp.2016.03.009

[25] Hodgkin J (1987) Sex determination and dosage compensation in Caenorhabditis elegans. Annu Rev Genet 21, 133–154. 3327460 10.1146/annurev.ge.21.120187.001025

[26] Chan SL , Yee KS , Tan KM and Yu VC (2000) The *Caenorhabditis elegans* sex determination protein FEM‐1 is a CED‐3 substrate that associates with CED‐4 and mediates apoptosis in mammalian cells. J Biol Chem 275, 17925–17928. 10764728 10.1074/jbc.C000146200

[27] Subauste MC , Sansom OJ , Porecha N , Raich N , Du L and Maher JF (2010) Fem1b, a proapoptotic protein, mediates proteasome inhibitor‐induced apoptosis of human colon cancer cells. Mol Carcinog 49, 105–113. 19908242 10.1002/mc.20594

[28] Begum SN , Ray AS and Rahaman CH (2022) A comprehensive and systematic review on potential anticancer activities of eugenol: from pre‐clinical evidence to molecular mechanisms of action. Phytomedicine 107, 154456. 36152592 10.1016/j.phymed.2022.154456

[29] Izadi M , Ali TA and Pourkarimi E (2021) Over fifty years of life, death, and cannibalism: a historical recollection of apoptosis and autophagy. Int J Mol Sci 22, 12466. 34830349 10.3390/ijms222212466 PMC8618802

[30] Su YC , Metzen LT , Velez LM , Bournique E , Seldin M , Buisson R , Kuo WW , Huang CY and Kaiser P (2023) Induction of resistance to oxaliplatin in cancer by a microRNA/Fem1B/Gli1 pathway. Am J Cancer Res 13, 6011–6025. 38187042 PMC10767360

[31] Timms RT , Mena EL , Leng Y , Li MZ , Tchasovnikarova IA , Koren I and Elledge SJ (2023) Defining E3 ligase‐substrate relationships through multiplex CRISPR screening. Nat Cell Biol 25, 1535–1545. 37735597 10.1038/s41556-023-01229-2 PMC10567573

[32] Manford AG , Rodriguez‐Perez F , Shih KY , Shi Z , Berdan CA , Choe M , Titov DV , Nomura DK and Rape M (2020) A cellular mechanism to detect and alleviate reductive stress. Cell 183, 46–61.e21. 32941802 10.1016/j.cell.2020.08.034

[33] Manford AG , Mena EL , Shih KY , Gee CL , McMinimy R , Martinez‐Gonzalez B , Sherriff R , Lew B , Zoltek M , Rodriguez‐Perez F *et al*. (2021) Structural basis and regulation of the reductive stress response. Cell 184, 5375–5390 e16. 34562363 10.1016/j.cell.2021.09.002 PMC8810291

[34] Chen X , Raiff A , Li S , Guo Q , Zhang J , Zhou H , Timms RT , Yao X , Elledge SJ , Koren I *et al*. (2024) Mechanism of psi‐pro/C‐degron recognition by the CRL2(FEM1B) ubiquitin ligase. Nat Commun 15, 3558. 38670995 10.1038/s41467-024-47890-5 PMC11053023

[35] Zhang W , Zha K , Xiong Y , Hu W , Chen L , Lin Z , Yu C , Zhou W , Cao F , Hu H *et al*. (2023) Glucose‐responsive, antioxidative HA‐PBA‐FA/EN106 hydrogel enhanced diabetic wound healing through modulation of FEM1b‐FNIP1 axis and promoting angiogenesis. Bioact Mater 30, 29–45. 37521275 10.1016/j.bioactmat.2023.07.006 PMC10382778

[36] Henning NJ , Manford AG , Spradlin JN , Brittain SM , Zhang E , McKenna JM , Tallarico JA , Schirle M , Rape M and Nomura DK (2022) Discovery of a covalent FEM1B recruiter for targeted protein degradation applications. J Am Chem Soc 144, 701–708. 34994556 10.1021/jacs.1c03980 PMC8928484

[37] Lee CE , Noh KM , Kim S , Hong J and Kim K (2025) Recent tumor necrosis factor‐related apoptosis‐inducing ligand engineering strategies for precise strike therapy against tumor. Biomater Res 29, 0170. 40110051 10.34133/bmr.0170 PMC11922527

[38] Luiz‐Ferreira A , Pacifico T , Cruz AC , Laudisi F , Monteleone G and Stolfi C (2023) TRAIL‐sensitizing effects of flavonoids in cancer. Int J Mol Sci 24, 16596. 38068921 10.3390/ijms242316596 PMC10706592

[39] Chen Y , Gu Y , Xiong X , Zheng Y , Liu X , Wang W and Meng G (2022) Roles of the adaptor protein tumor necrosis factor receptor type 1‐associated death domain protein (TRADD) in human diseases. Biomed Pharmacother 153, 113467. 36076575 10.1016/j.biopha.2022.113467

[40] Koliaki C and Katsilambros N (2022) Repositioning the role of tumor necrosis factor‐related apoptosis‐inducing ligand (TRAIL) on the TRAIL to the development of diabetes mellitus: an update of experimental and clinical evidence. Int J Mol Sci 23, 3225. 35328646 10.3390/ijms23063225 PMC8949963

[41] Jiang WJ , Xu CT , Du CL , Dong JH , Xu SB , Hu BF , Feng R , Zang DD , Meng XM , Huang C *et al*. (2022) Tubular epithelial cell‐to‐macrophage communication forms a negative feedback loop via extracellular vesicle transfer to promote renal inflammation and apoptosis in diabetic nephropathy. Theranostics 12, 324–339. 34987648 10.7150/thno.63735 PMC8690920

[42] Cartland SP , Patil MS , Kelland E , Le N , Boccanfuso L , Stanley CP , Cholan PM , Dona MI , Patrick R , McGrath J *et al*. (2024) The generation of stable microvessels in ischemia is mediated by endothelial cell derived TRAIL. Sci Adv 10, eadn8760. 39365855 10.1126/sciadv.adn8760 PMC11451529

[43] Seumois G , Ramirez‐Suastegui C , Schmiedel BJ , Liang S , Peters B , Sette A and Vijayanand P (2020) Single‐cell transcriptomic analysis of allergen‐specific T cells in allergy and asthma. Sci Immunol 5, eaba6087. 32532832 10.1126/sciimmunol.aba6087 PMC7372639

[44] Vanmeerbeek I , Naulaerts S , Sprooten J , Laureano RS , Govaerts J , Trotta R , Pretto S , Zhao S , Cafarello ST , Verelst J *et al*. (2024) Targeting conserved TIM3(+)VISTA(+) tumor‐associated macrophages overcomes resistance to cancer immunotherapy. Sci Adv 10, eadm8660. 39028818 10.1126/sciadv.adm8660 PMC11259173

[45] Gao W , Wang X , Zhou Y , Wang X and Yu Y (2022) Autophagy, ferroptosis, pyroptosis, and necroptosis in tumor immunotherapy. Signal Transduct Target Ther 7, 196. 35725836 10.1038/s41392-022-01046-3 PMC9208265

[46] Tong X , Tang R , Xiao M , Xu J , Wang W , Zhang B , Liu J , Yu X and Shi S (2022) Targeting cell death pathways for cancer therapy: recent developments in necroptosis, pyroptosis, ferroptosis, and cuproptosis research. J Hematol Oncol 15, 174. 36482419 10.1186/s13045-022-01392-3 PMC9733270

[47] Tang R , Xu J , Zhang B , Liu J , Liang C , Hua J , Meng Q , Yu X and Shi S (2020) Ferroptosis, necroptosis, and pyroptosis in anticancer immunity. J Hematol Oncol 13, 110. 32778143 10.1186/s13045-020-00946-7 PMC7418434

